# The effect of breast cancer on the Fourier transform infrared attenuated total reflection spectra of human hair

**DOI:** 10.3332/ecancer.2014.405

**Published:** 2014-02-20

**Authors:** Donald J Lyman, Sheila G Fay

**Affiliations:** 1 Department of Bioengineering and Department of Materials Science and Engineering, University of Utah, Salt Lake City, UT 84112, USA; 2 525 Lilly Rd NE, Olympia, WA 98506, USA

**Keywords:** human hair, breast cancer, signalling molecules, biomarkers, single fibre ATR-FT-IR spectra, variable angle ATR-FT-IR spectra, early detection, lipids

## Abstract

Changes in the Synchrotron x-ray diffraction pattern of scalp hair were shown to occur in patients with breast cancer. A preliminary Fourier transform infrared (FT-IR) spectroscopy study of scalp hair using attenuated total reflection (ATR) supported the concept that these changes are due to an increase in the lipid content of the hair fibre. This study was undertaken to determine whether the ATR-FT-IR spectrum obtained using a single hair fibre ATR cell could be used in the detection of breast cancer. In addition, variable angle ATR-FT-IR difference spectra were obtained to investigate the location and the molecular structure of this lipid material in the hair fibre, which appears to be an indicator of breast cancer. Patients with breast cancer showed an increase in the peak height ratio of the 1446–1456 cm^−1^ C–H bending absorption bands of the ATR-FT-IR spectra of a single hair fibre. Peak height ratios > 1.0 were indicative of breast cancer. The spectra of scalp hair of subjects with breast cancer also showed a slight shift in C–H bending absorption from 1446 to 1448 cm^−1^ and from 1456 to 1458 cm^−1^ that could result from the formation of secondary structures by the increased lipid material. Variable angle difference spectra indicated that this increased lipid material is located in the cuticle–cortex interface area and appears to be similar to the lipids normally found here. An alteration in hair biosynthesis in the follicle caused by breast cancer signalling molecules, or biomarkers, is most likely involved. ATR-FT-IR spectral analysis of a long hair fibre containing a distal portion formed when the breast cancer was present and a proximal portion formed after the breast cancer was removed showed that hair fibre synthesis had become normal after the removal of the cancer. This study demonstrates the potential of ATR-FT-IR analysis of a hair fibre in the early detection of breast cancer and in studying how hair acts as a biosensor for breast cancer.

## Introduction

Subtle changes have been observed in the alpha keratin x-ray diffraction pattern of hair from patients with breast cancer when x-rays were obtained using the Synchrotron high-brightness photon beam [1]. One or more rings of comparatively low intensity were superimposed at specific locations on the normal alpha keratin diffraction pattern. These rings were thought to arise from randomly oriented lipid bilayers in either the plasma membrane or in membranous inclusions in the hair cells [1]. Although some controversy surrounded the initial study due to the difficulty in detecting these low-intensity rings, it was reported that with careful attention to precise techniques, high-energy Synchrotron x-ray diffraction can reproducibly detect clear and consistent changes in the secondary structure of the scalp hair of breast cancer patients [2–6]. These studies have now advanced to clinical trials. Initial results confirm that an altered x-ray diffraction pattern indicates the presence of breast cancer with an overall accuracy of greater than 77% [7].

Since the changes observed in the x-ray diffraction patterns of hair from breast cancer patients suggest that compositional changes have occurred in the hair structure, it was of interest to determine the effect these changes might have on the infrared spectra of hair. Fourier transform infrared (FT-IR) spectroscopy, with its high resolution and high energy throughput over the entire spectral region and a good signal-to-noise ratio, enables the study of both the molecular (primary) and the conformational (secondary) structures of biological molecules. Attenuated total reflection (ATR) techniques can also allow the analysis of hair in a relatively non-destructive manner. An initial study of the complex ATR-FT-IR spectra of hair samples from non-cancer and cancer patients indicated that the spectra of hair from breast cancer patients showed increased C–H bending absorptions in the 1500–1400 cm^−1^ region compared with hair from non-cancer individuals [8]. This supported the postulate that the superimposed rings on the x-ray diffraction patterns were due to lipid materials. In addition, the effective depth of penetration (*d*_e_) of the evanescent wave of the infrared beam into the hair fibre suggested that at least some of this increased lipid material is located in the cuticle–cortex interface area [8, 9].

In this paper, we present the results using a single hair fibre ATR-FT-IR cell on hair samples from patients with and without breast cancer and where the breast cancer has been removed. A preliminary study using variable angle ATR-FT-IR difference spectroscopy was also conducted with these same hair samples to obtain additional information about the composition of the lipid materials, which appear to be indicators of breast cancer, and their location in the hair fibre.

## Methods

Samples of hair from 12 female Caucasian subjects were obtained by Sheila G. Fay. The subjects ranged in age from 37 to 85 and included both breast cancer positive and negative individuals. Eight strands of hair were cut close to the scalp from the bottom layer of hair on the nape of the scalp. Hair colours ranged from white, blonde, light brown, and red brown to dark brown. Each subject’s cancer status and other medical data pertinent to the study were also collected by Dr Fay.

This investigation was conducted as a double-blind study where only the generically coded hair samples were provided to the investigator for ATR-FT-IR analysis. The cancer status of the hair samples was disclosed to the investigator only after the conclusion of the ATR-FT-IR analysis. Consent forms and personally identifiable information were maintained solely by Dr Fay and not disclosed to the investigator. All forms and procedures comply with the requirements of Health Insurance Portability and Accountability Act, the Office of Human Research Protection at the U.S. Department of Health and Human Services, and Washington State law for patient protection and privacy.

Hair diameter was measured using a micrometer (Mitutoyo, Manual No. 1003) at the midpoint of the sample. Readings were made to the closest 0.01 mm (10 μm) and estimated to the closest 0.001 mm (1 μm). Hair diameters ranged from 38 to 53 μm.

ATR-FT-IR absorption spectra were obtained from 4000 to 700 cm^−1^ using 128 scans at a resolution of 4 cm^−1^ and Norton–Beer medium apodisation using a Thermo Nicolet Nexus 670 spectrometer with a liquid N_2_ cooled mercury–cadmium–telluride detector. The ATR cells used in this study were a SplitPea accessory (Harrick Scientific, Ossining, New York, United States) with a silicon (Si) and a zinc selenide (ZnSe) 45° internal reflection element (IRE) and a modified SeaGull variable angle reflection accessory (Harrick Scientific) with an ZnSe IRE. Variable angle spectra were obtained at various incident light angles (*θ*) from 60° to 42°.

The hair samples were used as received. Three strands from each subject were mounted on sample cards designed for the Seagull ATR cell and one strand from each subject was mounted on sample cards designed for the SplitPea ATR cell. The sample was positioned so that the portion of the hair, usually about 10 mm from the scalp end of the hair fibre, was over the hole in the sample card and aligned parallel to the IR beam. The pressure setting on the plunger of SplitPea ATR cell was set to the zero line on the pressure applicator scale. IREs were cleaned with methyl ethyl ketone before spectral collection of each hair sample.

Spectra manipulations, such as baseline corrections, water subtractions, curve fitting, second derivatives, and Fourier self-deconvolutions, were accomplished using the Grams 386 and Grams 32 programs (Galactic Industries, Salem, New Hampshire, United States).

## Results and discussion

The Harrick SplitPea ATR cell with its 250-μm diameter ‘hotspot’ on the hemispherical 45° IRE allows a spectrum to be obtained from a single hair fibre. The *d*_e_ of the evanescent wave of the 2000–700 cm^−1^ region of the ATR-FT-IR beam using *θ* = 45° and an ZnSe IRE is sufficient to obtain the spectrum of the cuticle and adjacent cortex regions of hair fibres even when the cuticle layer is about five to six cells thick [9]. Thus, one can obtain spectral information related to any changes in the molecular composition of the cuticle–cortex interface region giving rise to the increased C–H bending absorptions observed in hair from subjects with breast cancer [8].

**Figure 1.**
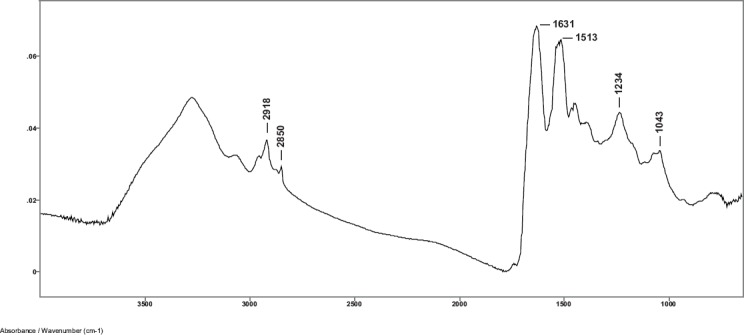
Baseline corrected and water subtracted ATR-FT-IR spectrum of a single human hair fibre using the SplitPea ATR cell.

Spectra were obtained from hair samples of the 12 subjects using the SplitPea ATR cell and were baseline corrected and water subtracted. A typical spectrum using minimum plunger pressure on the IRE is shown in Figure 1. Curve fitting of the Amide I region shows the presence of the alpha helical absorption at about 1652 cm^−1^. This indicates that the evanescent wave of the IR beam has penetrated beyond the cuticle into the alpha keratin material of the cortex region of the hair fibre in the 2000–700 cm^−1^ region of the spectrum [8, 9].

**Figure 2.**
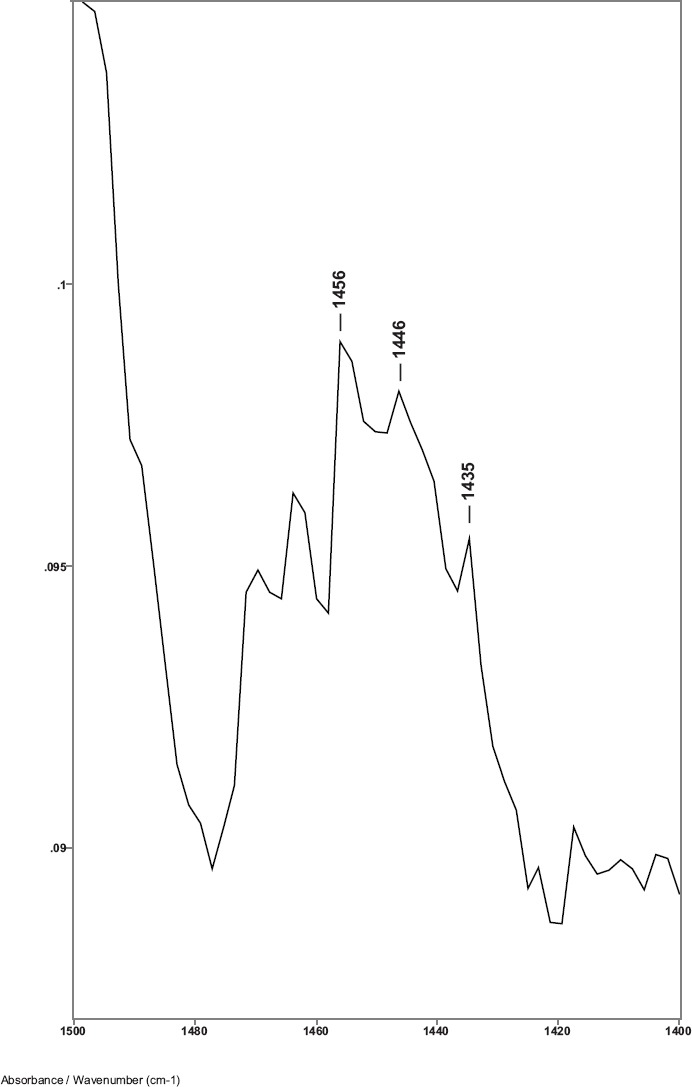
The 1480–1420 cm^−1^ region of ATR-FT-IR spectrum of hair from a non-breast cancer patient (sample 010).

**Figure 3.**
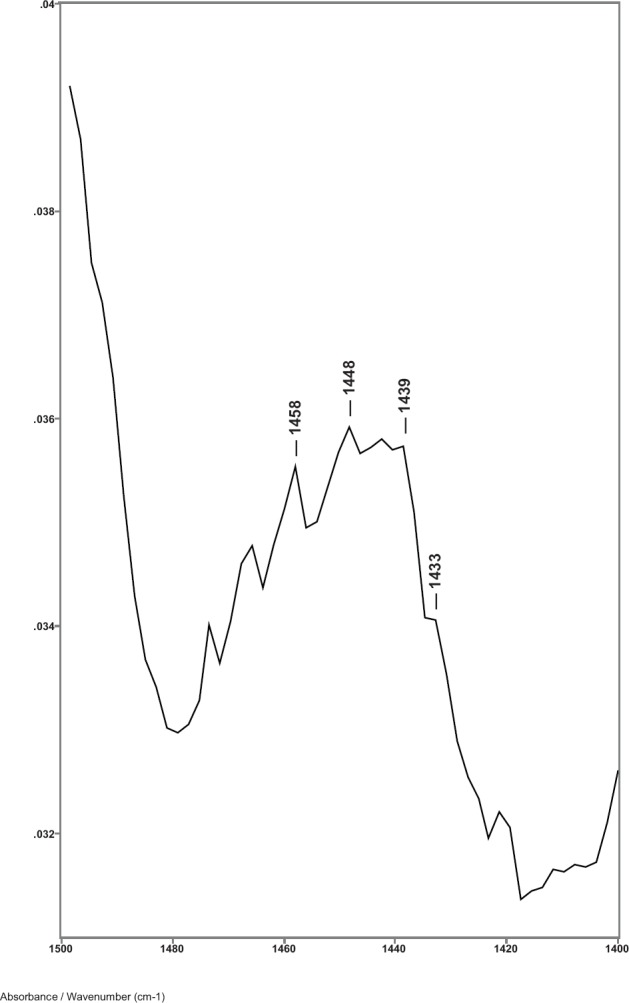
The 1480–1420 cm^−1^ region of ATR-FT-IR spectrum of hair from a breast cancer patient (sample 008).

**Figure 4.**
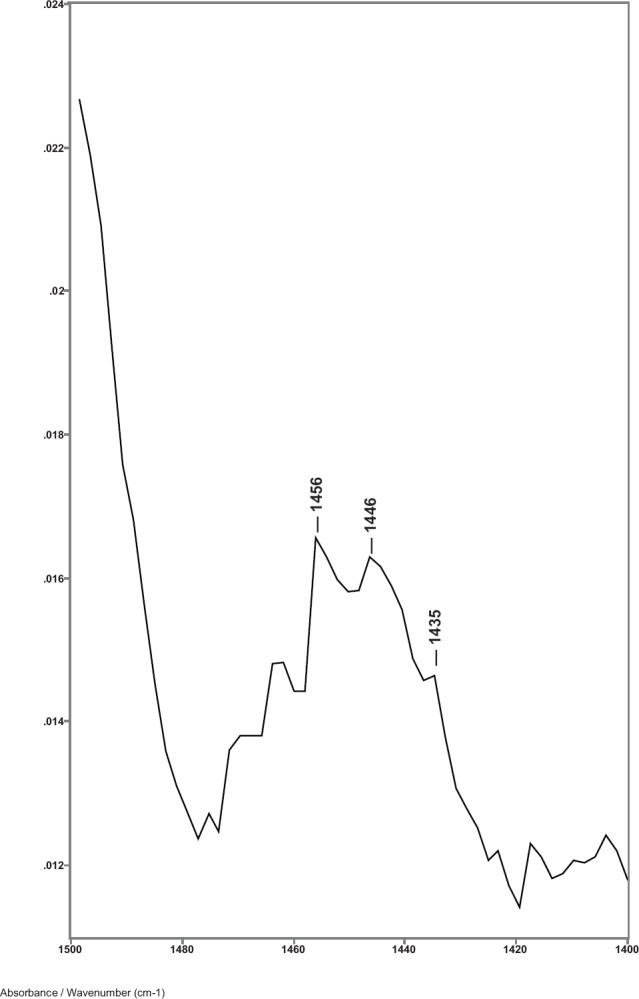
The 1480–1420 cm^−1^ region of ATR-FT-IR spectrum of hair from a patient that had breast cancer removed (sample 003).

While previous studies showed that the C–H bending absorptions around 1446 and 1437 cm^−1^ increased in intensity in the presence of breast cancer, it was the 1446–1456 cm^−1^ peak height ratios that consistently gave values > 1.0 for cancer [8]. Thus, in this study, the relative amount of lipid material in the cuticle–cortex interface region was determined from the ratio of the peak heights of the C–H bending absorption (-CH_2_-) around 1446 cm^−1^ to the C–H bending absorption (-CH_3_) around 1456 cm^−1^. Figure 2 shows this 1500–1400 cm^−1^ region of spectra for hair sample 010 from a non-cancer patient. Hair sample 010 showed a 1446/1456 cm^−1^ peak height ratio of 0.991. Figure 3 shows this region for hair sample 008 from a cancer patient. Hair sample 008 showed a 1448/1458 cm^−1^ peak height ratio of 1.012. Figure 4 shows this region for hair sample 003 from patient whose cancer had been surgically removed. Hair sample 003 showed a 1446/1456 cm^−1^ peak height ratio of 0.984. The peak ratios for all hair samples are tabulated in Table 1. The slight shift in the C–H bending absorption peaks from 1446 to 1448 cm^−1^ and 1456 to 1458 cm^−1^ in cancer subjects is most likely due to changes in the secondary structure of the lipid resulting from the increased amount of lipid content present rather than from the formation of a different lipid material. This is consistent with the observation of the low-intensity rings in the x-ray diffraction patterns in the hair of cancer subjects, which indicated changes in the secondary structure of what James *et al* [1] proposed to be lipid material.

Sample numbers 001, 006, 008, 009, and 011A showed peak height ratios greater than 1.010 and were considered positive for breast cancer. Sample numbers 001, 006, and 008 were later confirmed to be from breast cancer subjects, and thus were correctly identified as positive. Patient 001 had an infiltrating ductal carcinoma, patient 006 had an infiltrating ductal and lobular carcinoma, and patient 008 had an infiltrating ductal/bilateral carcinoma.

Sample 009 may be considered a false positive based on the subject’s medical history of non-detectable breast cancer. However, James [10] reported changes in the x-ray diffraction pattern of hair observed months before malignancies were clinically detected, and it is reasonable to expect ATR-FT-IR analysis to also provide early indications of breast cancer. Though it is possible that sample 9 may not be a false positive but an early indication of cancer, this could not be confirmed since follow-up medical data on this subject were not available.

In reviewing the cancer status, it was found that sample 011A was from a subject who had breast cancer (ductal carcinoma *in situ*) surgically removed just 60 days prior to the hair sample being obtained for this study. Thus, the sample was clinically classified as non-cancer. Since hair grows at around 0.35 mm/day, we calculated that about 21 mm of the proximal portion of the hair fibre was formed after the cancer was surgically removed, while the more distal portion of the hair fibre had been formed before the cancer was removed. In measuring the hair fibre mounted on the sample card, we determined that the original spectrum for sample 011A was of a section of the hair approximately 26 mm from the proximal end. The sample had been inadvertently positioned on the sample card so that an area more distal on the hair fibre than the standard 10 mm was exposed to the IR beam. This distal area had been formed prior to the surgical removal of the cancer. Thus, the hair sample of this subject provided a good internal control for our study. The first spectrum (011A) of this sample had shown a C–H bending peak ratio of 1.046 indicating cancer. A second spectrum (011B) was then obtained from the portion of the same hair fibre at about 10 mm from the proximal end. This portion of the hair fibre that had been formed after the cancer was surgically removed showed a C–H bending peak ratio of 0.983 indicating non-cancer. These results are consistent with our findings and the x-ray diffraction studies reported by James [5] that, once the breast cancer is removed, the structure of the hair fibre returns to normal. This underscores the importance of the precision needed in targeting the region on the hair fibre as close to the proximal end as practicable for obtaining ATR-FT-IR spectra.

Spectra of eight subjects showed C–H bending peak height ratios of 1.000 or less and were considered negative for breast cancer. All of these subjects were clinically classified as non-cancer, and thus were correctly identified by ATR-FT-IR analysis. Of these eight subjects, five had been previously diagnosed with breast cancer and successfully treated 29 months to seven years prior to the collection of samples for this study. Again, these findings are consistent with the x-ray diffraction studies showing that, once the cancer is removed, the structure of the hair’s new growth returns to normal [5].

The peak height ratios indicative of breast cancer ranged from 1.012 to 1.046 with an average of 1.025; the peak height ratios indicative of no breast cancer ranged from 0.974 to 1.000 with an average of 0.987. The increase of C–H bending absorption intensities supports the hypothesis that the observed rings on the x-ray diffraction patterns of hair from subjects with breast cancer are due to increase in lipids [1, 8].

While the peak height ratios for the 1435/1456 cm^−1^ C–H bending absorptions were also generally higher for hairs from patients with breast cancer (see Table 1), there was some scatter in the data. The peak height ratios for the cancer patients ranged from 0.952 to 1.019 with an average of 0.989; the peak height ratios indicative of non-cancer patients ranged from 0.878 to 0.978 with an average of 0.932. Up-field shifts (to 1439 cm^−1^) were usually observed in cancer hairs.

The increase in lipid material in the hair fibre appears to start when the breast cancer is formed and stops when the breast cancer is removed [4, 5]. This suggests that signalling molecules, or biomarkers, from the developing breast cancer are being expressed into the blood and carried to the papilla of the hair follicle where they modify the hair biosynthesis in the fibroblasts, resulting in increased lipid material being formed. Thus, the hair fibre acts as a selective chemical sensor for certain breast cancer signalling molecules, enabling early and accurate detection of breast cancer.

One such signalling molecule may be fatty acid synthase (FASN), an enzyme that catalyses the final steps in the biosynthesis of long-chain fatty acids [11]. However, the cells of colon, prostate, and lung cancers have also been shown to release FASN, though its concentration may vary depending on the type of cancer. While colon cancer was reported to be detectable by x-ray diffraction analysis of a hair fibre [12], the superimposed rings on the x-ray diffraction pattern were in a slightly different position than those found in breast cancer. This would suggest that more than one signalling molecule is involved in changing the hair biosynthesis. Thus, information on the type of lipids formed and their location in the hair fibre structure may provide clues to the cancer signalling pathway involved in the process of modifying the hair biosynthesis. This information might be obtained from variable angle ATR-FT-IR spectra using difference spectroscopy.

The calculated depth of penetration (*d*_p_) of the evanescent wave of the IR beam into the hair fibre is dependent on the refractive indexes of the IRE and the sample, the frequency of the IR beam, and the incident light angle of the IR beam [13]. However, the actual, or effective depth of penetration (*d*_e_) of the evanescent wave increases as the ratio of the refractive indexes of the sample-to-IRE approaches 1.0 [8, 9, 13–15]. For hair/ZnSe, *d*_e_ was shown to be 3 *d*_p_ [8, 15]. Spectra using an ZnSe IRE with *θ* = 45° shows the helical Amide I absorptions from the keratin in cortex cells, thus the *d*_e_ of the evanescent wave of the IR beam was sufficient to penetrate into the cortex region of these hair fibres in the region between 2000 and 700 cm^−1^. This would indicate that the cuticle thickness is about five to six cuticle cells in thickness, i.e., about 2.6–3.1 μm in thickness.

Variable angle spectra of hair from these subjects were obtained from *θ* = 43° to 54° using the Harrick SeaGull variable angle ATR-FT-IR cell with an ZnSe IRE. Subtractions of the various *θ* spectra to obtain spectral slices (difference spectra) were done using the Grams Subtract AB Auto-Factor program with *X* axis limits of 1500–1400 cm^−1^. The C–H bending peak height ratios of 1446-8/1456-8 cm^−1^ bands of the difference spectra of the 12 samples all showed an increase in this C–H bending absorption ratio occurring in either the 47º minus 48º spectral slice or the 48º minus 49º spectral slices with peak intensities being higher in the cancer subjects. This indicates that the increased concentration of lipid materials occur at a depth of about 3.2–3.5 μm in the hair fibre, and supports our assumption that the increased lipid material is located in the cuticle–cortex interface area. This area is comprised of the cuticle–cortex cell membrane complex (CMC) and the flattened orthocortical cells adjacent to the cuticle.

A little is known about the cuticle–cortex CMC other than it appears to be similar to that proposed for the CMC between cuticle–cuticle and cortex–cortex cells, i.e., a delta layer sandwiched between two lipid beta layers [16, 17]. Recently, Robbins [18] proposed three different structures for CMCs indicating distinct differences between the cuticle–cuticle, cortex–cortex, and cuticle–cortex CMCs based on a number of published electron microscopy and enzymatic digestion studies. The proposed structure of delta layer in the cuticle–cortex CMC is quite distinct. It contains three free fatty acids (possibly palmitic, stearic, and oleic acids), cholesterol (or cholesterol sulphate) and possibly ceramides. On the cuticle side of the delta layer, the lipids are bound via hydrophobic globular proteins, while on the cortex side of the delta layer, the lipids are bound via hydrophilic globular proteins. Wolfram [19] had earlier suggested that saccharides, not globular proteins, may act as the adhesive binders for lipids.

The similarities of the peak positions of the C–H bending absorptions in hair from both breast cancer subjects and non-cancer subjects suggest that the type of additional lipid material being formed has not changed. Thus, the presence of biomarkers from breast cancer appears to result in an increase in the amount of lipid materials normally present in the delta layer of the cuticle–cortex CMC and in the flattened orthocortical cells adjacent to the cuticle, but not in the type of lipid material formed. This is supported by increases in peak heights of absorption bands associated with lipids at, for example, 1739–1740 cm^−1^ (lipid ester), 1240 cm^−1^ (C–H lipid), 1163 cm^−1^ (C–O stretch lipid), 1161 cm^−1^ (C–O–C stretch lipid), and 1055 cm^−1^ (cholesterol) in the hairs from breast cancer patients.

Studies on lipid rafts [20] suggest that increased interactions between palmitic acid, cholesterol and/or ceramides (lipids normally found in the cuticle–cortex CMC) and cortical cells may be involved in producing the secondary structures giving rise to the x-ray diffraction rings as well as the IR band frequency shifts in the hairs from breast cancer patients. A more detailed difference spectroscopy analysis of the cuticle–cortex interface area of hair fibres is in progress to determine more precisely the composition and structure of this interface area.

## Conclusion

The ATR-FT-IR spectra of a single hair fibre obtained using the Harrick Scientific SplitPea ATR cell with an ZnSe IRE (*θ* = 45°) can be used to detect breast cancer in Caucasian women. Hair from subjects with breast cancer showed an increase in the peak height ratio of the 1446–1456 cm^−1^ C–H bending absorptions and a slight shift in absorptions from 1446 to 1448 cm^−1^ and 1456 to 1458 cm^−1^. Peak height ratio values > 1.0 were indicative of breast cancer.

Difference spectra indicate that this increased lipid material forms in the cuticle–cortex interface region and may be due to breast cancer signalling molecules affecting the biosynthesis of lipid material in the fibroblasts involved in forming the cuticle–cortex CMC and the adjacent orthocortical cells. This increased amount of lipid material results in a change in the lipid secondary structure (also observed in the x-ray diffraction patterns) causing a slight shift in the C–H bending absorptions. Lipid rafts may be involved.

This study demonstrates the potential for ATR-FT-IR spectroscopy, not only in the early detection of breast cancer, but in providing clues to the molecular and signalling processes involved in how hair acts as a biosensor in the detection of breast cancer.

## Conflicts of interest

The authors have no conflicts of interest to declare.

## Figures and Tables

**Table 1.** ATR-FT-IR peak height ratios of selected C–H bending vibrations of hair used to detect breast cancer.

Sample no.	Peak (cm^−1^)	Peak ratio^a^	Peak (cm^−1^)	Peak ratio^b^	Medical^c^/IR^d^
011A^e^	1446	1.046	1437	0.981	C C
006	1448	1.031	1439	0.952	C C
001	1448	1.023	1439	0.985	C C
009	1448	1.014	1439	1.019	NC C
008	1448	1.012	1439	1.006	C C
005	1446	1.000	1435	0.978	NC NC
004	1446	0.994	1435	0.911	CR NC
010	1446	0.991	1435	0.965	NC NC
007	1446	0.985	1435	0.957	NC NC
011B^e^	1446	0.983	1437	0.971	CR NC
002	1446	0.984	1437	0.878	CR NC
003	1446	0.984	1435	0.885	CR NC
012	1446	0.974	1435	0.912	CR NC

a Peak height ratios of the 1446–1448 cm^−1^ peak to the 1456–1458 cm^−1^ peak.

b Peak height ratios of the 1435–1439 cm^−1^ peak to the 1456–1458 cm^−1^ peak.

c From patient medical forms: C indicates patient has breast cancer, CR indicates patient had breast cancer surgically removed, and NC indicates that patient did not have breast cancer.

d Based on ATR-FT-IR analysis: C indicates cancer and NC indicates non-cancer.

e 011A from distal end of hair sample 011 and 11B from proximal end of hair sample 011.

## References

[1] James V (1999). Using hair to screen for breast cancer. Nature.

[2] Meyer P, James VJ (2001). Experimental confirmation of a distinctive diffraction pattern in hair from women with breast cancer. J Natl Cancer Inst.

[3] James VJ (2003). The traps and pitfalls inherent in the correlation of changes in the fibre diffraction pattern of hair with breast cancer. Phys Med Biol.

[4] James VJ (2005). Early diagnosis of breast cancer by hair diffraction. Int J Cancer.

[5] James VJ (2006). A place for fiber diffraction in the detection of breast cancer?. Cancer Detect Prev.

[6] Corino GL, French PW (2008). Diagnosis of breast cancer by X-ray diffraction of hair. Int J Cancer.

[7] Corino GL (2009). Characterization of a test for invasive breast cancer using X-ray diffraction of hair – Results of a clinical trial. Breast Cancer Basic Clin Res.

[8] Lyman DJ, Murray-Wijelath J (2005). Fourier transform infrared attenuated total reflection analysis of human hair: comparison of hair from breast cancer patients with hair from healthy subjects. Appl Spectrosc.

[9] Lyman DJ, Schofield P (2008). Attenuated total reflection Fourier transform spectroscopy analysis of human hair fiber structure. Appl Spectrosc.

[10] James VJ (2003). False-positives in studies of changes in fibre diffraction of hair from patients with breast cancer may not be false. J Natl Cancer Inst.

[11] Little JK, Kridel SJ (2008). Fatty acid synthases are expressed in cancer. Subcell Biochem.

[12] James VJ (2003). Fibre diffraction from a single hair can provide an early non-invasive test for colon cancer. Med Sci Monit.

[13] Harrick NJ (1985). Internal Reflection Spectroscopy.

[14] Mirabella FM (1983). Strength of interaction and penetration of infrared radiation for polymer films in internal reflection spectroscopy. J Polym Sci (Polym Phys Ed).

[15] Mirabella FM, Harrick NJ (1985). Internal Reflection Spectroscopy: Review and Supplement.

[16] Feughelman M (1997). Mechanical Properties and Structure of Alpha-Keratin Fibres.

[17] Robbins CR (2002). Chemical and Physical Behavior of Human Hair.

[18] Robbins CR (2009). The cell membrane complex: three related but different cellular cohesion components of mammalian hair fibers. J Cosmet Sci.

[19] Wolfram LJ (2003). Human hair: a unique physiochemical composite. J Am Acad Dermatol.

[20] Lingwood D, Simons K (2010). Lipid rafts as a membrane-organizing principle. Science.

